# Whole-Exome Sequencing of Discordant Monozygotic Twin Families for Identification of Candidate Genes for Microtia-Atresia

**DOI:** 10.3389/fgene.2020.568052

**Published:** 2020-10-22

**Authors:** Xinmiao Fan, Lu Ping, Hao Sun, Yushan Chen, Pu Wang, Tao Liu, Rui Jiang, Xuegong Zhang, Xiaowei Chen

**Affiliations:** ^1^Department of Otolaryngology, Peking Union Medical College Hospital, Beijing, China; ^2^Chinese Academy of Medical Sciences and Peking Union Medical College, Beijing, China; ^3^Bioinformatics Division, BNRIST and MOE Key Laboratory of Bioinformatics, Department of Automation, Tsinghua University, Beijing, China; ^4^Department of Otolaryngology, The Ohio State University, Columbus, OH, United States; ^5^Department of Otolaryngology Head and Neck Surgery, Beijing Tiantan Hospital, Capital Medical University, Beijing, China; ^6^Annoroad Gene Technology (Beijing) Co., Ltd, Beijing, China

**Keywords:** microtia-atresia, whole-exome sequencing, monozygotic discordant twins, genes, bioinformatics analysis

## Abstract

**Objective:**

We used data from twins and their families to probe the genetic factors contributing to microtia-atresia, in particular, early post-twinning variations that potentially account for the discordant phenotypes of monozygotic twin pairs.

**Methods:**

Six families of monozygotic twins discordant for congenital microtia-atresia were recruited for study. The six patients shared a consistent clinical phenotype of unilateral microtia-atresia. Whole-exome sequencing (WES) was performed for all six twin pairs and their parents. Family segregation and multiple bioinformatics methods were applied to identify suspicious mutations in all families. Recurring mutations commonly detected in at least two families were highlighted. All variants were validated via Sanger sequencing. Gene Ontology (GO) analysis was performed to identify candidate gene sets and related pathways. Copy number variation (CNV), linkage analysis, association analysis and machine learning methods were additionally applied to isolate candidate mutations, and comparative genomics and structural modeling tools used to evaluate their potential roles in onset of microtia-atresia.

**Results:**

Our analyses revealed 61 genes with suspected mutations associated with microtia-atresia. Five (*HOXA4, MUC6*, *CHST15*, *TBX10*, and *AMER1*) contained 7 *de novo* mutations that appeared in at least two families, which have been previously reported as pathogenic for other diseases. Among these, *HOXA4* (c.920A>C, p.H307P) was determined as the most likely pathogenic variant for microtia-atresia. GO analysis revealed four gene sets involving 11 pathways potentially related to underlying pathogenesis of the disease. CNVs in three genes (*UGT2B17*, *OVOS*, and *KATNAL2*) were detected in at least two families. Linkage analysis disclosed 13 extra markers for the disease, of which two (*FGFR1* and *EYA1*) were validated via machine learning analysis as plausible candidate genes for the disease.

**Conclusion:**

Based on comprehensive genetic and bioinformatic analyses of WES data from six families of discordant monozygotic twins with microtia-atresia, we identified multiple candidate genes that may function in post-twinning onset of the disease. The collective findings provide novel insights into the pathogenesis of congenital microtia-atresia.

## Introduction

Microtia-atresia is a rare congenital condition characterized by malformation of the external ear accompanied by atresia of the external ear canal. The severity of the condition varies, from only a mild dysmorphogenesis to the complete absence of the external ear and ear canal. The reported prevalence of microtia-atresia in different countries ranges from 0.83 to 17.4 per 10,000 births (Deng et al., 2016; Cabrejo et al., 2019). Similar to many other structural birth defects, microtia-atresia can occur as either an isolated symptom or as part of a congenital syndrome. A set of abnormalities, including vertebral anomalies, craniofacial anomalies, renal abnormalities, cardiac defects, holoprosencephaly, and polydactyly, are associated with microtia-atresia (Bartel-Friedrich, 2015; Stoll et al., 2016). The effectiveness of prenatal ultrasonography in diagnosis of this condition is low due to high phenotypic heterogeneity.

The external ear is derived from the first two branchial arches along with the first branchial groove in between, with embryonic development beginning from week 5 that lasts several months during the fetal stages (Luquetti et al., 2012). During this period, six hillocks surround the first pharyngeal cleft, each of which differentiates into a specific structure of the pinna through growth, fusion, and morphogenesis, finally migrating from an initially low position to the normal anatomical location (Luquetti et al., 2012; Georgakopoulos and Zafar Gondal, 2020). Any risk factors that influence this developmental process could trigger microtia-atresia.

Congenital microtia-atresia is considered a multifactorial developmental malformation induced by both environmental and genetic factors. Environmental factors including retinoic acid intake, viral infection and anemia during pregnancy, and maternal intrinsic factors such as multiple pregnancy and diabetes, have been highlighted in earlier epidemiological studies (Lee et al., 2012; Liu et al., 2018). A number of experts have successfully identified microtia-atresia susceptibility genes using a variety of approaches, and a certain degree of success achieved mainly for syndromic microtia-atresia (Cox et al., 2014; Gendron et al., 2016; Wang et al., 2019; Lu et al., 2020). However, the genes responsible for non-syndromic microtia-atresia are yet to be determined and validated. Lu et al. (2020) introduced a gene pool composed of all suspected microtia-atresia candidate genes identified from five main approaches. Although our understanding of the risk factors of microtia-atresia has developed, more specific pathogenic mechanisms remain to be fully elucidated. Comprehensive analysis of the etiology of microtia-atresia should therefore provide a solid basis for further mechanistic insights.

Microtia has a familial clustering rate of about 3–34%, and both autosomal-dominant and autosomal-recessive patterns of inheritance have been reported (Kelley and Scholes, 2007; Chafai Elalaoui et al., 2010; Gejje et al., 2017). Compared with sporadic cases, these pedigrees of microtia-atresia hold great value for genetic exploration. Twins, notably, have proved to be of significance when studying and separating the genetic and environmental factors, thanks to the fact that monozygotic twins have nearly identical genetic materials (van Dongen et al., 2012; Zhang et al., 2016).

While several earlier genetic studies on microtia-atresia have been conducted in Chinese populations, the majority have involved sporadic cases or simple analysis. The complex etiology of this disease is yet to be fully elucidated. In the current study, whole-exome sequencing (WES) was performed on six Chinese monozygotic twin pairs discordant for microtia-atresia along with their parents, and multiple bioinformatics approaches were employed to determine the genetic factors contributing to post-twinning onset of the disease.

## Materials and Methods

### Ethics

Written informed consent was obtained from the individual(s) AND/OR minor(s)’ legal guardian/next of kin for the publication of any potentially identifiable images or data included in this article.

### Family Recruitment

Monozygotic twins hospitalized at the Department of Otolaryngology, Peking Union Medical College Hospital (PUMCH), between May 2014 and May 2019 showing discordance for congenital microtia-atresia were enrolled for study. Family history was obtained and clinical features of all the study subjects and their parents evaluated via review of medical records. Patients diagnosed with syndromic microtia-atresia or with a positive family history were excluded. Twins and their parents finally included for study were subjected to WES. This study was approved by the institutional review board of PUMCH (JS-796) and a written informed consent was provided by legal guardian of each twins. Authorization was also obtained from the guardians for disclosure of recognizable individuals in photographs and collection of blood for future analyses.

### Library Construction and Exome Sequencing

Peripheral blood was taken and total genomic DNA was extracted. The DNA was broken into ∼250 bp fragments using a TIANamp Blood DNA Kit (Tiangen, Beijing, China). DNA quality was confirmed via gel electrophoresis prior to library construction. Following end repair and A-tailing steps, a pair of sequencing adaptors was attached to both sides of the fragments. DNA of all subjects was tagged by amplifying adaptor-bonded products using index-tagged primers. The amplified products were purified using the QIAquick PCR Purification kit (QIAGEN). Sonication (cat: FB705, Thermo Fisher, Waltham, MA, United States) and hybrid capture with xGen Exome Research Panel v1.0 (Integrated DNA Technologies, Inc., Coralville, IA, United States) were employed to enrich and sequence genomic DNA on the Illumina HiSeq 2500 platform with read lengths of 125 bp, providing ∼386x coverage depth across all samples. Raw image files were processed using base-calling software (Illumina 1.7) with default parameters.

### Variant Identification and Annotation

After initial quality-filtration, raw sequencing alignments produced by the Illumina pipeline were then subjected to data pre-processing. Low quality sequences and sequencing adaptors were removed before the read mapping step. After raw data were performed by the Fastqc v0.18.1 tool, high quality pair-ended reads of each sample were processed using the Burrows-Wheeler Aligner (BWA) 0.7.8-r455 (with default parameters) package to the GRCh37/hg19 reference genome. We performed base quality score recalibration together with single nucleotide variants (SNV) and short indels using a Genome Analysis Toolkit (GATK 3.8) package based on the improved BAM (.bam) files. For subsequent analysis, high quality and reliable mutations were obtained after filtering and screening using SAMtools 1.6.

Sequence variants, including SNVs and small insertions or deletions (InDels), were annotated using ANNOVAR software^1^ and categorized as missense, nonsense or splice-site mutations, along with other genomic features. For coding or splice-site mutations, the conservatism of the variant site and the predicted effect on protein function were evaluated using the *in silico* tools SIFT^2^, PolyPhen-2^3^, MutationTaster^4^, and CADD^5^. Sequenced reads were collected, filtered for quality, and aligned to the human reference sequence (UCSC Genome Browser hg19^6^) using the Burrows-Wheeler Aligner. Genotype calling was performed using GATK.

### Novel Variant or CNV Identification and Recurrence Analysis in Twin Families

Family segregation was systematically performed to screen *de novo* variants and CNVs. The following criteria were used: (1) inclusion of protein-altering variants only including frameshift, InDels, missense, stopgain, and intron-exon boundary mutations, (2) application of the 1000 Genomes Project, the HapMap CHB (Han Chinese in Beijing, China) population, the National Heart, Lung, and Blood Institute Exome Sequencing Project (ESP), dbSNP (v.144), and the Exome Aggregation Consortium (ExAC) Browser for minor allele frequency (MAF) checking, whereby common variants present at a frequency of >10% in at least one of the above databases were excluded, and (3) filtering out of synonymous and intron region mutations. CNVs were identified using open source software called CNVkit, a tool kit to infer and visualize copy number from targeted DNA sequencing data. Burrows Wheeler Alignment tool was employed for the alignment of sequencing data to the Human Reference Genome (hg 19) to generate bam files as input. Normal reference used for CNV identification were constructed using sequencing data from the healthy individuals in the same family with option “batch method amplicon” and the Agilent SureSelectXT All Exon Kit 51 Mb bed file. The CNV of the affected probands were estimated using the segment file by “call” command with the “filter ci” option of the CNVkit. Other default CNVkit settings were used for CNV identification. CNV recurrences were estimated in unrelated families. For the identified variants, recurrence analysis was conducted to determine mutations common to at least two families, which were likely to be pathogenic.

The identified mutations were validated via polymerase chain reaction (PCR) amplification and Sanger sequencing. Relevant sequences were amplified from the twins and their family members and the fragments subsequently purified using an Agencourt AMPure XP kit (Beckman Coulter, United States). Sanger sequences were processed using the ABI3730xl DNA Sequencer (Applied Biosystems/Thermo Fisher Scientific, United States) and the results analyzed using Sequencing Analysis 5.2 software (Applied Biosystems/Thermo Fisher Scientific). The Human Splicing Finder program^7^ was applied to evaluate the strength of ectopic splicing sites. The ConSeq server was used to analyze conservatism of amino acids and HOPE server^8^ used to predict structural variations.

### Molecular Analysis

The STRING website was used to predict the relationships of proteins with known microtia-atresia-associated pathways, including Wnt, Bmp, Fgf, RA and Endothelin-1^9^. HGVS nomenclature guidelines^10^ were applied to label the identified mutations. All mutations were cross-referenced to the Human Gene Mutation Database (HGMD) to determine whether they were classified as pathogenic from earlier studies. We applied the online software PSIPRED (v3.3) to analyze secondary structural variations associated with potentially pathogenic mutations of candidate genes, SWISS-MODEL to predict the tertiary structures of the encoded proteins, and the Research Collaboratory for Structural Bioinformatics Protein Data Bank (RCSB PDB)^11^ to search for three-dimensional (3D) structures. PyMOL software was employed to reconstruct the wild-type and mutant proteins.

### Analysis of GO Pathway Enrichment

All the potential pathogenic genes from each family and individual pathways were determined separately and the recurrent pathways in at least two families and gene sets included in each pathway identified. The statistical significance of the enrichment pathway (*P*) was determined using the hypergeometric test formula:

$$P=1-\sum_{i=0}^{m-1}\frac{\frac{M}{i}\,\frac{N-M}{n-i}}{\frac{N}{n}}$$

where *M* represents the gene number in each pathway, *m* the potential pathogenic gene number in each pathway, *N* the gene number in all pathways, and *n* the potential pathogenic gene number in *N*. Pathways with *q*-values < 0.2 and *p*-values < 0.05 were considered significantly enriched.

### Linkage Analysis, Correlation Analysis and Mathematical Modeling

In order to identify the common SNPs that are associated with increased risk of microtia-atresia, plink analysis was performed. To ensure data quality, sample threshold for Hardy-Weinberg equilibrium, 0.001 (Fisher’s exact test), mutations without missing call rate > 75% and SNP call rate > 95% were set in the control cohort. Genotype data for Utah Residents (CEPH) with Northern and Western European Ancestry (CEU), Han Chinese in Beijing (CHB), and Southern Han Chinese (CHS) populations from the 1KG Project were extracted. Principal component analysis (PCA) was performed on these samples together with the genotyped samples using smartPCA software. A gene-based test comparing the burden of identified variants in cases and controls was performed using SKAT-O implemented in the SKAT package that included only non-synonymous variants. Bonferroni correction was applied to account for multiple testing. For the markers identified through correlation analysis, support vector machine (SVM) combined with random forest modeling analysis was performed to detect the most likely pathogenic gene variants.

### Statistical Analysis

All data were analyzed using SPSS (V 21, the International Business Machines Corp) software, and unpaired *t*-tests were used to analyze all data sets. Statistical significance was defined as a *p*-value < 0.05.

## Results

### Demographics and Clinical Characteristics

**FIGURE 1:**
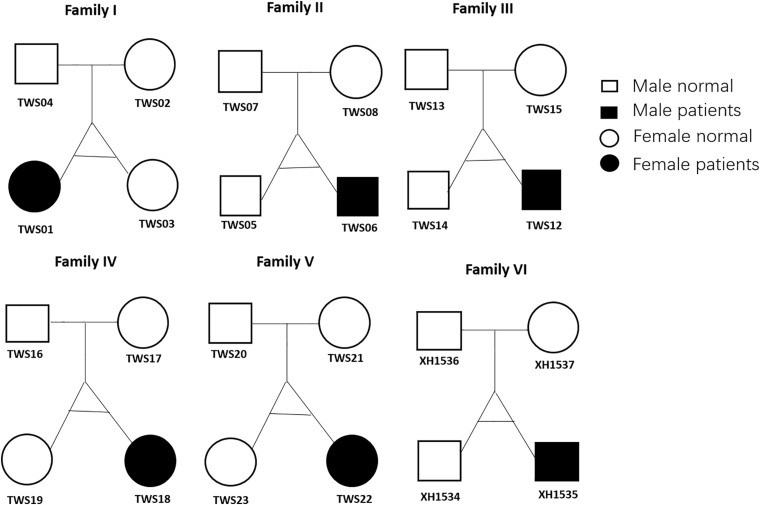
Twin family pedigrees. Symbols indicate a female (circle) or male (square) family member; The text next to the symbols represents the family member ID. Whole-exome sequencing was performed for all 24 members.

A total of 24 subjects including six monozygotic twins showing discordance for congenital microtia-atresia and their parents were enrolled for study (Figure 1). Analysis of consanguinity revealed that all six twins were monozygotic (as each co-twin all shared 99.9% similar SNVs) and none of the parents were born to consanguineous families. Detailed demographic information of this cohort is presented in Supplementary Table 1. All six patients (3 males, 3 females; 4 right, 2 left; mean age, 12.6 years; range, 6.5–23 years) presented with a consistent clinical phenotype classified as grade III (Hunter Classification) (Hunter et al., 2009) external ear deformity. Pure-tone audiometry tests disclosed unilateral moderate to severe conductive hearing loss. No deformities of other systems were detected in the patient group and all the other subjects in this cohort were normal. All parents denied exposure to known environmental risk factors during pregnancy.

### Sequencing Quality

A total of 3,675,683 bases were captured and sequenced in this study. About 98.86% of the clean reads could be mapped to the target regions after alignment to the reference human genome (GRCH37/hg19). The average targeted region depth was 387.6-fold and 93.66% was covered by at least 20 reads, indicative of high quality of sequencing.

### Novel Variant or CNV Identification and Recurrent Analysis in Twin Families

A total of 178 non-synonymous *de novo* variants of 86 genes were identified after family segregation and Esp6500 and 1000 Genomes public database filtering (Supplementary Table 2). Recurrence analysis in different families revealed that five genes (*HOXA4, MUC6*, *CHST15*, *TBX10*, *AMER1*) including seven recurring heterozygous mutations (Table 1) in at least two families. These seven mutations included two frameshift variants, c.1366delC (p.R456fs) and c.1356_1357insGCCC (p.P453fs), in carbohydrate sulfotransferase 15 (*CHST15*) and five missense variants, c.920A>C (p.H307P) in homeobox A4 (*HOXA4*), c.4553C>T (p.T1518I) and c.5705C>T (p.T1902I) in mucin 6 (*MUC6*), c.791C>T (p.P264L) in T-box transcription factor 10 (*TBX10*), and c.61C>T (p.R21C) in APC membrane recruitment protein 1 (*AMER1*). All the mutations were predicted as pathogenic with the Condel program. CNVs of UDP glucuronosyltransferase family 2 member B17 (*UGT2B17*) and alpha-2-macroglobulin like 1 pseudogene (*OVOS*) were detected in four families and katanin catalytic subunit A1 like 2 (*KATNAL2*) in two families (Table 2).

**TABLE 1:** *De novo* variants recurrent in at least two families.

Genes	Family I	Family II	Family III	Family IV	Family V	Family VI
HOXA4		c.920A > C:p.H307P	c.920A > C:p.H307P		c.920A > C:p.H307P	
MUC6		c.4553C > T:p.T1518I c.5705C > T:p.T1902I		c.5705C > T:p.T1902I	c.4553C > T:p.T1518I	
CHST15	c.1366delC:p.R456fs		c.1356_1357insGCCC:p.P453fs			c.1366delC:p.R456fs
TBX10	c.791C > T:p.P264L					c.791C > T:p.P264L
AMER1			c.61C > T:p.R21C		c.61C > T:p.R21C	

**TABLE 2:** CNVs recurrent in at least two families.

Genes	Recurrent subjects	Related disease
UGT2B17	TWS01| TWS06| TWS18| TWS12	Osteoporosis
OVOS	XH1535| TWS06| TWS18| TWS22	Unknown
KATNAL2	TWS06| TWS12	Autism

### Molecular Analysis of Identified Variants

**FIGURE 2:**
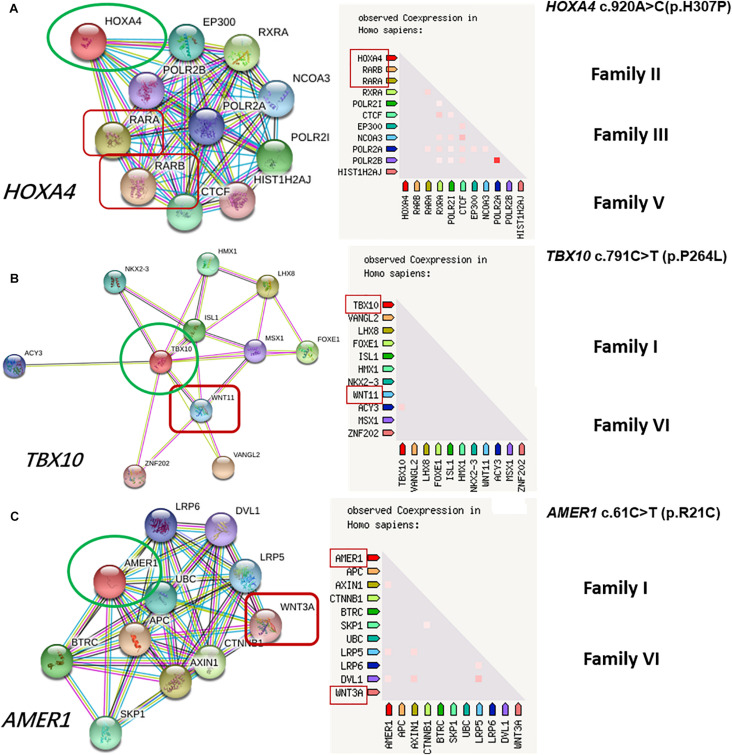
Protein-to-protein interactions of candidate genes with known microtia-atresia associated pathways. **(A)** HOXA4 directly interacts with RARA of the RA pathway, detected in three families (II, III, V); **(B)** TBX10 interacts directly or indirectly with WINT11 of the Wnt pathway; **(C)** AMER1 interacts indirectly with WINT3A of the Wnt pathway. Both genes were detected in Families I and VI.

Prediction of protein-protein interactions of the eight genes (*HOXA4, MUC6*, *CHST15*, *TBX10*, *AMER1, UGT2B17, OVOS, KATNAL2*) was conducted using online STRING software. Five pathways (Wnt, Bmp, Fgf, RA and Endothelin-1) closely related to craniofacial development (Jia et al., 2016) were ultimately selected. According to the interaction network, HOXA4 interacts directly with RARA of the RA pathway, TBX10 directly or indirectly with WINT11 of the Wnt pathway, and AMER1 indirectly with WINT3A of the Wnt pathway (Figure 2). Our results highlight *HOXA4, TBX10* and *AMER1* as potential high-risk candidate pathogenic genes of microtia-atresia that exert their effects through disrupting the RA and Wnt signaling pathways.

**FIGURE 3:**
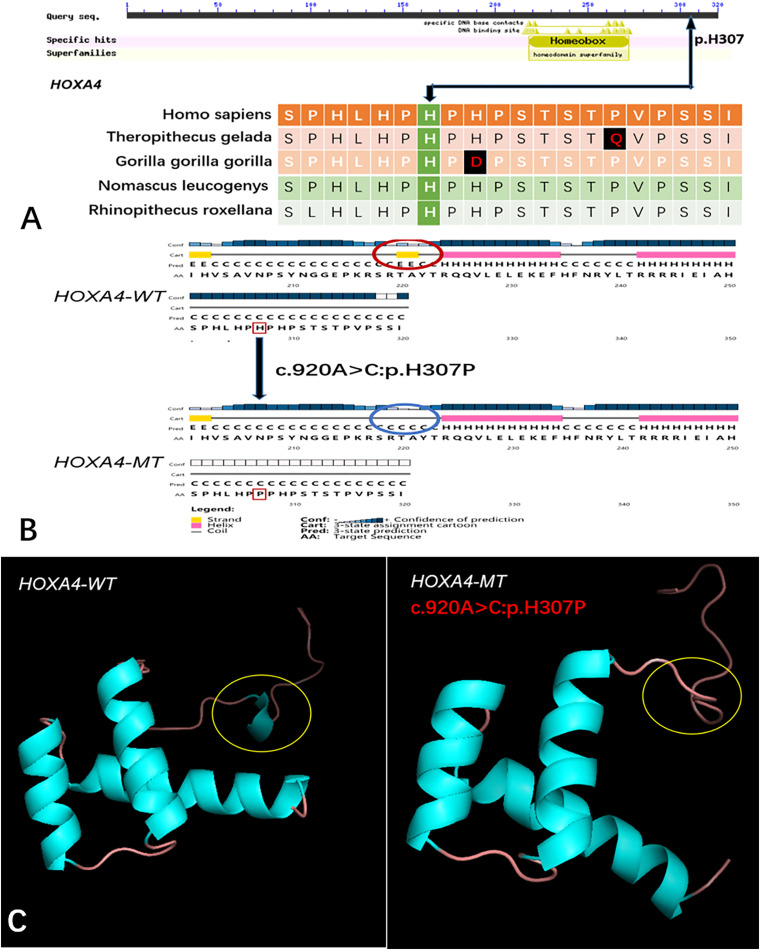
Conservation of the *HOXA4* mutation site across different species and wild-type and mutant secondary and crystal structures. **(A)** Amino acids affected by the *HOXA4 c.920A* > *C:p.H307P* mutation are highly conserved among different species; **(B)** The *HOXA4 c.920A* > *C:p.H307P* mutation identified in this study is predicted to modify the local secondary structure of the protein, circled in red and blue; **(C)** Template-based modeling of tertiary structures. Global view of the crystal structures of wild-type and mutant proteins demonstrating changes in protein structure attributable to the substitution. Local homology prediction demonstrating a high confidence rate (73.49%). WT, wild-type; MT, mutant.

**FIGURE 4:**
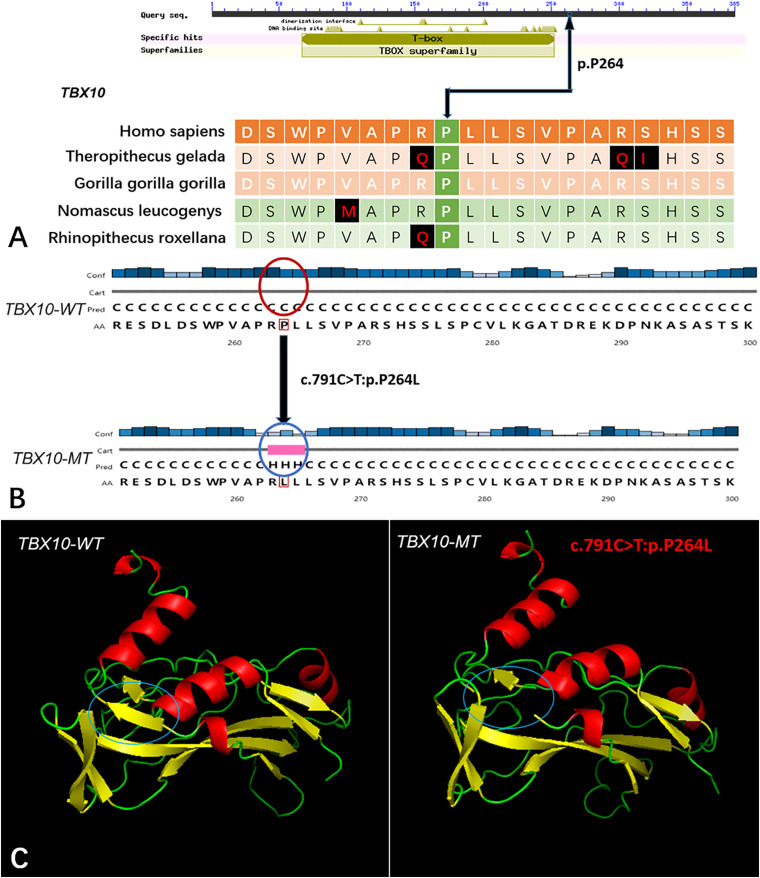
The conservation of *TBX10* mutation site in different species, and wild-type and mutated-type secondary and crystal structures. **(A)** The amino acid affected by the mutation (*TBX10* c.791C >T:p.P264L) was highly conserved among different species; **(B)** The mutation (TBX10 c.791C >T:p.P264L) was predicted to modify the local secondary structure of the protein, which were circled as red and blue; **(C)** Template-based modeling of the tertiary structures was carried out. Global view of the crystal structures of wild-type and mutated-type proteins demonstrated the change of protein structure brought by the amino acid substitution. Local homology prediction demonstrated high confidence rate (81.62%). WT means wild-type; MT means mutated-type.

**FIGURE 5:**
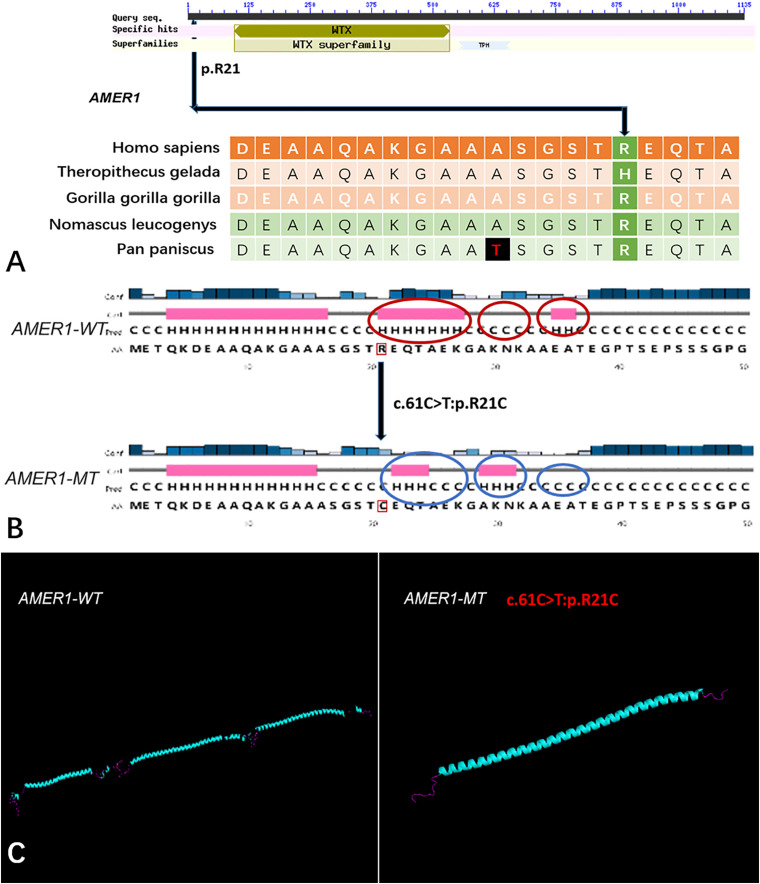
The conservation of *AMER1* mutation site in different species, and wild-type and mutated-type secondary and crystal structures. **(A)** The amino acid affected by the mutation (*AMER1* c.61C > T:p.R21C) was relatively conserved among different species; **(B)** The mutation (*AMER1* c.61C > T:p.R21C) was predicted to modify the local secondary structure of the protein, which were circled as red and blue; **(C)** Template-based modeling of the tertiary structures was carried out. Global view of the crystal structures of wild-type and mutated-type proteins demonstrated the change of protein structure brought by the amino acid substitution. Local homology prediction demonstrated high confidence rate (69.52%). WT means wild-type; MT means mutated-type.

Five genes (*HOXA4, MUC6*, *CHST15*, *TBX10*, *AMER1*) incorporating seven mutations were identified in at least two families. The *HOXA4(c.920A* > *C, p.H307P)* substitution was commonly detected in three families, indicating a causative role in microtia-atresia. Histidine at position 307 was mutated to proline, which was predicted to be “damaging” by both Polyphen-2 and SIFT. The mutation took place in a site showing high conservatism across different species. The wild-type residue was positively charged whereas the mutant residue was non-polar and therefore more hydrophobic causing a significant secondary structural alteration validated by the HOPE server structural analysis. Further examination of the crystal structure showed that the mutant protein was significantly different from its wild-type counterpart (Figure 3). The mutation is located within a downstream area annotated in UniProt as a regulatory region. The domain originally binds to DNA as monomers, homodimers and/or heterodimers to exert transcriptional regulation of vital biological processes, which are likely to be affected by the mutation. Our collective data suggest that the mutation is deleterious and support a contributory role in microtia-atresia. The mutation site, c.791C > T:p.P264L in *TBX10*, is conserved across different species and predicted to be “Damaging” by SIFT and “Benign” by Polyphen-2. The secondary and crystal structures of both wild-type and mutant proteins are shown in Figure 4. For *AMER1*, one missense mutation (c.61C > T:p.R21C) was detected in two families and predicted as relatively conserved in different species and “Damaging” both by SIFT and Polyphen-2. The secondary structures of the mutant and its respective wild-type counterpart are presented in Figure 5.

Two recurrent mutations of *MUC6* (c.5705C > T:p.T1902I, c.4553C > T:p.T1518I) were identified. The p.T1902I substitution was predicted as “Possibly Damaging” by Polyphen-2 and “Tolerated” by SIFT, while p.T1518I was classified as “Tolerated” by both Polyphen-2 and SIFT. The secondary and tertiary structural changes between wild-type and mutant proteins are depicted in Supplementary Figures 1, 2. STRING results revealed no direct or indirect protein-protein interactions with the five known pathways. Moreover, the amino acid residues affected by both mutations were not conserved among different species. Two mutations (c.1366delC:p.R456fs, c.1356_1357insGCCC:p.P453fs) in *CHST15* were predicted as “Damaging” by both Polyphen-2 and SIFT. Both were frameshift mutations, which could generate truncated proteins owing to a premature stop codon or disrupted amino acid sequence and consequently result in abnormal function.

### Analysis of GO Pathway Enrichment

Four gene sets involved in 11 pathways were potentially related to pathogenesis of congenital microtia-atresia (Supplementary Table 3). Among these, epidermal cell differentiation and epidermis development pathways may play crucial roles in auricle development, since both are associated with cell development and differentiation and auricular formation relies on normal development, differentiation, and migration of cells. Both pathways are linked to the downstream Wnt pathway, which is reported to be related to microtia-atresia^12^.

### Linkage and Correlation Analyses and Mathematical Modeling

Linkage analysis revealed 27 markers potentially related to the disease, which were further narrowed down to 13 based on data from correlation analysis (Supplementary Table 4). Among the markers identified based on correlation analysis, SVM combined with Random Forest modeling analysis showed that two candidate genes, fibroblast growth factor receptor 1 (*FGFR1*) and EYA transcriptional coactivator and phosphatase 1 (*EYA1*), play important roles in pathogenesis of microtia-atresia. Both genes are related to deformities in maxillofacial development (Wang et al., 2018; Shah et al., 2020).

## Discussion

Microtia-atresia is a complex disease, with numerous genetic and environmental factors or epigenetic alterations potentially involved in disease pathogenesis. Although several studies on the etiology and pathogenesis of microtia-atresia have been conducted, no consensus has been reached to date. Most microtia-atresia cases are sporadic, with 3–34% cases being clustered in families (Genc et al., 2012). Several gene sequencing and bioinformatics analyses on genetic and chromosomal variants related to microtia-atresia have been documented. Pedigree studies demonstrate both autosomal-dominant and autosomal-recessive patterns of inheritance (Brown et al., 2013; Piceci et al., 2017; Chen et al., 2018; Lupo et al., 2019; Kini et al., 2020; Lu et al., 2020). However, the crucial candidate genes in sporadic cases are yet to be identified. Determination of the contributory factors in discordant twins with the condition should help gain a better understanding of the underlying causes.

Monozygotic twins develop from a single fertilized egg carrying identical sets of genetic information and thus provide a considerable advantage in determining the genetic epidemiology of complex traits and diseases. A series of potential confounding factors including but not limited to age, gender, *in utero* and early growth environment and other maternal and cohort effects are all naturally excluded in the study of twins (Boomsma et al., 2002; van Dongen et al., 2012). As intra-individual genetic variations have been observed and CNV analyses in monozygotic twins have demonstrated somatic mosaicism, the utility of discordant MZ twin models in resolving differences in genetic constitution has been proved effective (Bruder et al., 2008; O’Huallachain et al., 2013). While a number of previous studies have focused on the etiology of monozygotic twins discordant for syndromic microtia-atresia (Verona et al., 2006; Prasad et al., 2013; Chen et al., 2018), no twin studies on genetic detection have been conducted for non-syndromic microtia-atresia. To explore the underlying etiology of microtia-atresia in this study, WES was performed on six monozygotic twin pairs discordant for non-syndromic microtia based on the hypothesis that all the affected twins should show causative *de novo* variations.

*HOX* genes encode a set of proteins that have a conserved homeodomain of 60 amino acids, carrying a vital role of building and maintaining the sensorimotor circultry of vertebrates. Both mutations in *HOXA1* or *HOXA2* have been reported to result in microtia-atresia (Qiao et al., 2015; Piceci et al., 2017). Homeobox protein HOXA4 serves to locate cells on the anterior-posterior axis. This transcription factor regulates the related biological processes by binding to the 5′-flanking sequence of the coding region with various affinities. HOXA4 is associated with various cancer types and its downregulation leads to inhibition of growth and invasion of lung cancer cells (Cheng et al., 2018). Based on molecular analyses, the *HOXA4* (c.920A>C:p.H307P) mutation may acquire pathogenicity by disrupting the DNA binding process during the early embryonic period and thus inhibit development of auricle cartilage. CHST15 catalyzes the chemical reaction that produces rare E-disaccharide units, a novel mediator of local fibrosis. Mutations in this gene are reported to be involved in metastasis and invasion of cancer (Ito et al., 2017). In this study, two frameshift mutations of the gene were identified, which may exert pathogenic activity by disrupting the formation of extracellular matrix crucial for cartilage development. Further animal studies will be conducted to validate the pathogenicity of all seven mutations.

At least two recurrent mutations of different candidate genes were commonly identified in families, except family IV. In addition, GO enrichment analysis led to the detection of four gene sets potentially related to pathogenesis of congenital microtia-atresia. We suspect that these genes act concertedly to cause the disease, suggesting that microtia-atresia is a polygenic process. While our findings provide novel insights in this field, further studies are required to establish the mechanisms underlying the pathogenesis of microtia-atresia.

In terms of CNVs, three genes (*UGT2B17, OVOS*, and *KATNAL2*) were identified in at least two families. The *UGT2B17* gene encodes a member of the uridine diphosphoglucuronosyltransferase protein family, which catalyzes glucuronidation, an important intermediate step in steroid metabolism. Copy number variations of this gene are associated with susceptibility to osteoporosis (Yang et al., 2008; Uddin et al., 2013). To our knowledge, no reports on diseases related to copy number variations in other genes are documented in the literature. Linkage and mathematical modeling analyses further revealed two candidate genes (*FGFR1* and *EYA1*). Earlier studies have shown an association between *EYA1* and microtia-atresia (Wang et al., 2018). However, the candidate mutation in *EYA1* was only identified in one family and has not been characterized as pathogenic to date. Further investigations on larger cohorts with sequencing technology and animal models should be conducted for validation of these findings.

Our study has several limitations that should be taken into consideration. For example, coverage is considered a critical index for the quality of WES. According to some researchers, an average coverage of > 500x is need for optimal detection of low-frequency SNVs. However, this strategy is not very cost-effective and is consequently not performed routinely (Spencer et al., 2014). The exome region constitutes only 1–2% of the entire genome, and WES inevitably leaves out the non-coding mRNA elements and/or intronic or intergenic regulatory regions that may be responsible for the discordant phenotypes of twins. Moreover, phenotypic discordance of monozygotic twins may be caused by post-zygotic somatic mutations which may occur at a rate of ∼1.2/10^–7^ mutations per base pair (Li et al., 2014). Above all, existing data of twins suggest that CNVs or exome DNA differences are extremely rare. Thus, we must consider the possibility that the phenotypic discordance among the twins are not directly gene-related but are caused by epigenetic or environmental factors. Perhaps, by increasing the sample sizes of MZ discordant microtia-atresia twins, we would be able to identify more genetic contribution of the disease among post-zygotic variations.

## Conclusion

Whole-exome sequencing combined with various bioinformatics methods was conducted on six discordant monozygotic twins with congenital microtia-atresia and their parents, with a view to identifying potential pathogenic genes. The results obtained significantly improved our understanding of the genetic pathogenesis of the disease. We identified several genes, in particular, *HOXA4* with the same mutation (*c.920A* > *C, p.H307P*), commonly shared by three families, which may be deleterious and contribute to microtia-atresia.

## Data Availability Statement

The datasets generated for this study can be found in NCBI SRA accession PRJNA665131.

## Ethics Statement

The studies involving human participants were reviewed and approved by the institutional review board of PUMCH (JS-796). Written informed consent to participate in this study was provided by the participants’ legal guardian/next of kin.

## Author Contributions

XC conceived and designed this experiment. XF collected the DNA samples, analyzed the data, and composed the manuscript. LP composed the manuscript. HS analyzed the data. YC, PW, TL, and RJ helped collect the patients’ information and analyzed the data. XZ ascertained the data analysis and manuscript polish. All the authors read and approved the final manuscript.

## Disclaimer

The authors alone are responsible for the content and writing of this paper.

## Conflict of Interest

TL was employed by the company Annoroad Gene Technology. The remaining authors declare that the research was conducted in the absence of any commercial or financial relationships that could be construed as a potential conflict of interest.

## Footnotes

1 http://annovar.openbioinformatics.org

2 http://sift.jcvi.org/

3 http://genetics.bwh.harvard.edu/pph2/

4 http://www.mutationtaster.org/

5 http://cadd.gs.washington.edu/

6 http://genome.ucsc.edu/

7 http://www.umd.be/HSF/

8 http://www.cmbi.ru.nl/hope/

9 https://string-db.org

10 http://www.hgvs.org/mutnomen

11 https://swissmodel.expasy.org/

12 http://geneontology.org/

## Supplementary Material

Supplementary Figure 1 The wild-type and mutated-type secondary structures of *MUC6 mutations.***(A)** The mutation (*MUC6*c.5705C > T:p.T1902I) was predicted to modify the local secondary structure of the protein, which were circled as red and blue; **(B)** The mutation (*MUC6* c.4553C > T:p.T1518I) was predicted to modify the local secondary structure of the protein circled as red and blue. WT means wild-type; MT means mutated-type.

Supplementary Figure 2 The wild-type and mutated-type crystal structures of MUC6 mutations. **(A1)** Global view of the crystal structures of wild-type proteins of MUC6; **(A2)** Global view of the mutated-type (*MUC6*c.5705C > T:p.T1902I) proteins. The change of protein structure brought by the amino acid substitution was circled. Local homology prediction demonstrated high confidence rate (86.13%). **(B2)** Global view of the mutated-type (*MUC6* c.4553C > T:p.T1518I) proteins. The crystal structure was obviously different between the wild-type and mutated-type. Local homology prediction demonstrated high confidence rate (79.32%). WT means wild-type; MT means mutated-type.
