# Supplementary figures and images for: Whole-Exome Sequencing of Discordant Monozygotic Twin Families for Identification of Candidate Genes for Microtia-Atresia
Source: Front Genet. 2020 Oct 22;11:568052. doi: 10.3389/fgene.2020.568052 (PMC7642525; doi:10.3389/fgene.2020.568052)

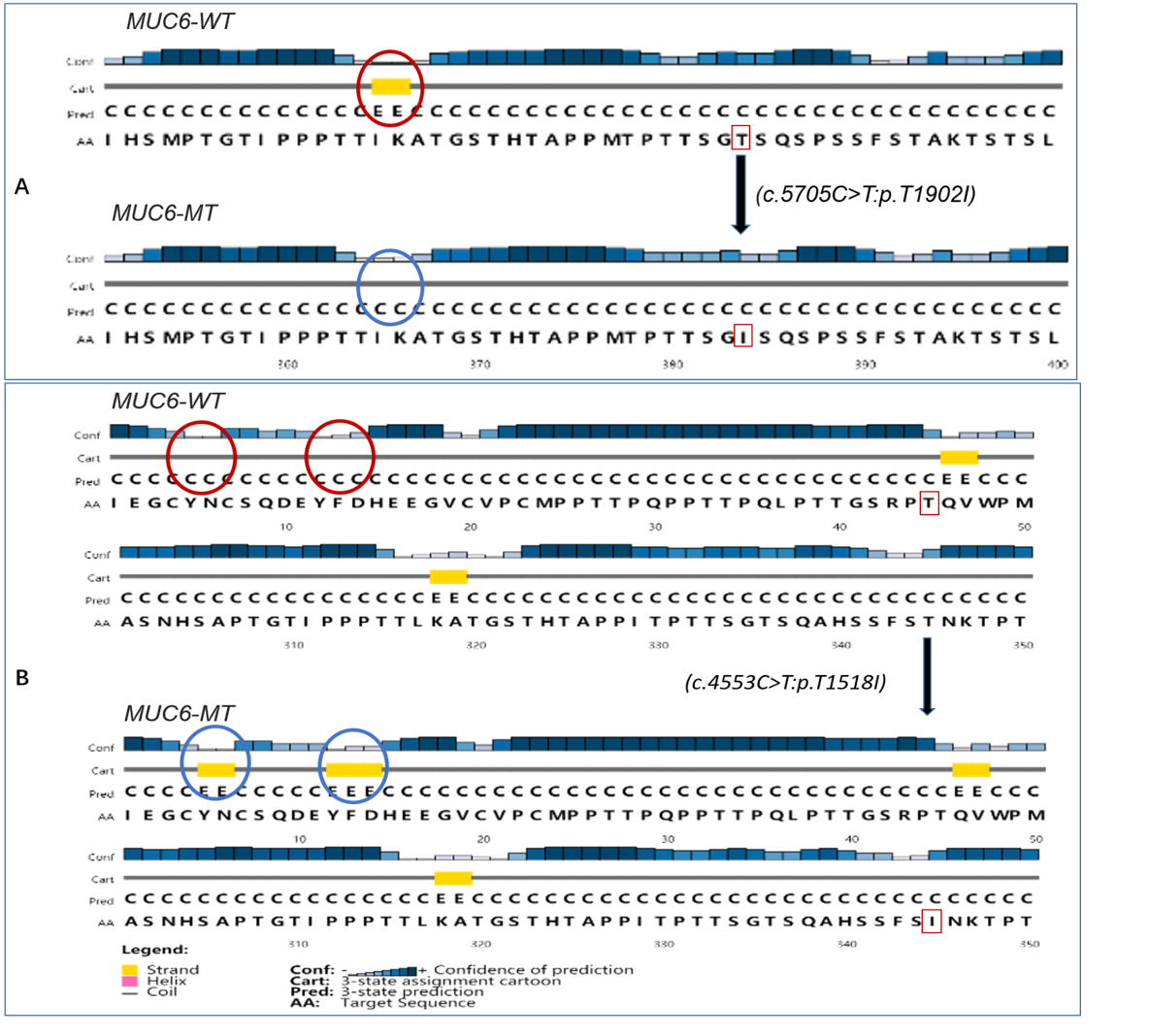

Supplement: Supplementary Figure 1 — The wild-type and mutated-type secondary structures of MUC6 mutations.(A) The mutation (MUC6c.5705C > T:p.T1902I) was predicted to modify the local secondary structure of the protein, which were circled as red and blue; (B) The mutation (MUC6 c.4553C > T:p.T1518I) was predicted to modify the local secondary structure of the protein circled as red and blue. WT means wild-type; MT means mutated-type. [file Image_1.TIF]

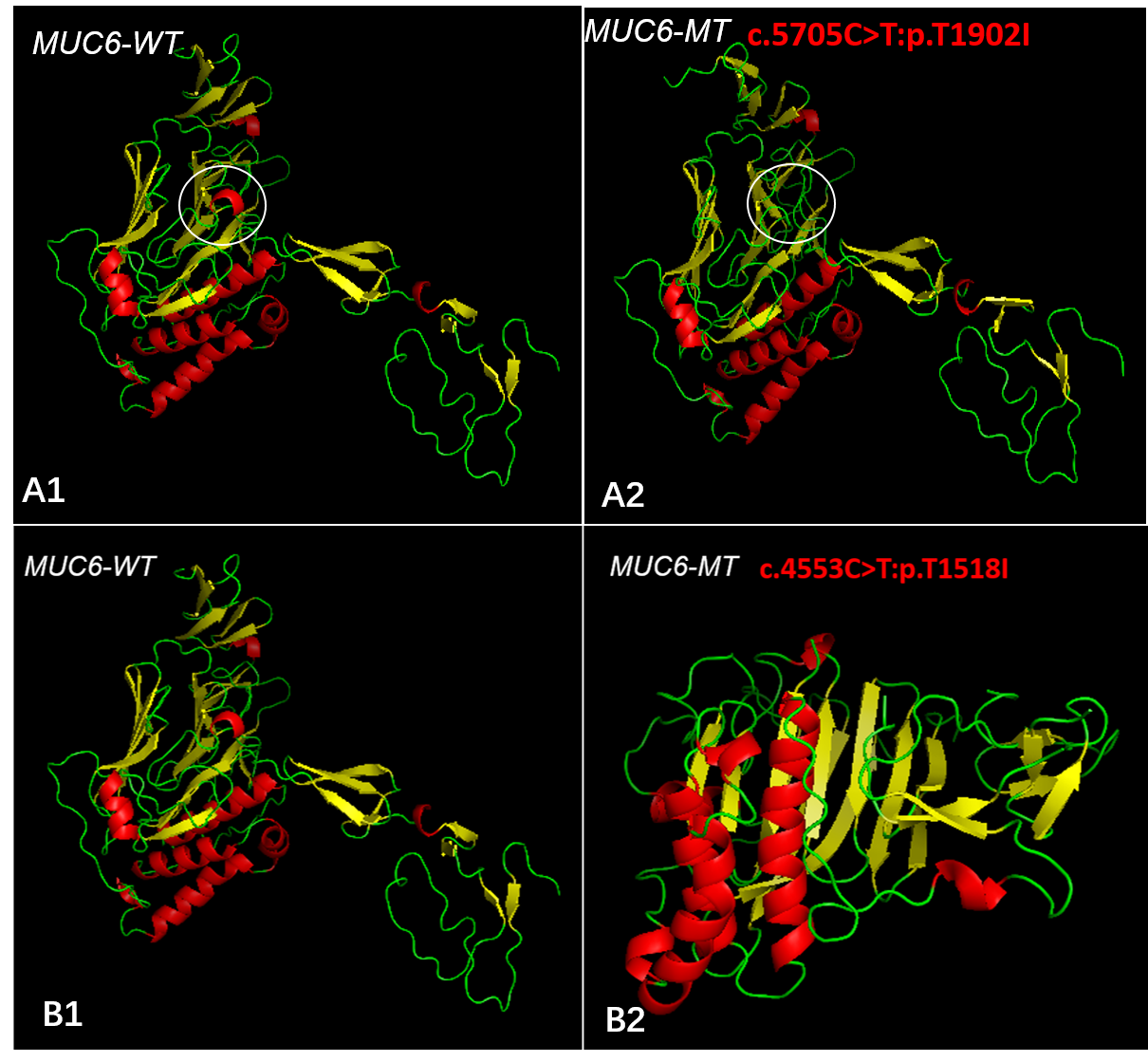

Supplement: Supplementary Figure 2 — The wild-type and mutated-type crystal structures of MUC6 mutations. (A1) Global view of the crystal structures of wild-type proteins of MUC6; (A2) Global view of the mutated-type (MUC6c.5705C > T:p.T1902I) proteins. The change of protein structure brought by the amino acid substitution was circled. Local homology prediction demonstrated high confidence rate (86.13%). (B2) Global view of the mutated-type (MUC6 c.4553C > T:p.T1518I) proteins. The crystal structure was obviously different between the wild-type and mutated-type. Local homology prediction demonstrated high confidence rate (79.32%). WT means wild-type; MT means mutated-type. [file Image_2.TIF]
