# Supplementary material for: Whole-Exome Sequencing of Discordant Monozygotic Twin Families for Identification of Candidate Genes for Microtia-Atresia
Source: Front Genet. 2020 Oct 22;11:568052. doi: 10.3389/fgene.2020.568052 (PMC7642525; doi:10.3389/fgene.2020.568052)
Supplement: Supplementary file 3 [file Table_1.DOCX]

**Supplementary Table 1. Demographics and clinical data of patients**

| **Item** | **Gender*** | | **Age (years)** | | **Affected side** | **Degree of microtia**^&^ | | | **Mean air conduction hearing threshold of affected ear(dB HL)** |
| --- | --- | --- | --- | --- | --- | --- | --- | --- | --- |
| **TWS01** | | F | 6.5 | right | | III | | 60 | |
| **TWS02** | | F | 31 |  | |  | |  | |
| **TWS03** | | F | 6.5 |  | |  | |  | |
| **TWS04** | | M | 33 |  | |  | |  | |
| **TWS05** | | M | 8 |  | |  | |  | |
| **TWS06** | | M | 8 | left | | III | | 65 | |
| **TWS07** | | M | 31 |  | |  | |  | |
| **TWS08** | | F | 31 |  | |  | |  | |
| **TWS12** | | M | 23 | right | | III | | 55 | |
| **TWS13** | | M | 50 |  | |  | |  | |
| **TWS14** | | M | 23 |  | |  | |  | |
| **TWS15** | | F | 48 |  | |  | |  | |
| **TWS16** | | M | 41 |  | |  | |  | |
| **TWS17** | | F | 41 |  | |  | |  | |
| **TWS18** | | F | 7 | left | | III | | 55 | |
| **TWS19** | | F | 7 |  | | |  |  | |
| **TWS20**  **TWS21**  **TWS22**  **TWS23**  **XH1536**  **XH1537**  **XH1534**  **XH1535** | | M  F  F  F  M  F  M  M | 55  56  23  23  32  32  8  8 | right  right | | | III  III | 65  70 | |

*F, female; M, male; Age, age in years when diagonosed as microtia-atresia;

^&^Degrees of auricular dysplasias were evaluated according to Hunter’s classification;
