# Supplementary material for: Whole-Exome Sequencing of Discordant Monozygotic Twin Families for Identification of Candidate Genes for Microtia-Atresia
Source: Front Genet. 2020 Oct 22;11:568052. doi: 10.3389/fgene.2020.568052 (PMC7642525; doi:10.3389/fgene.2020.568052)
Supplement: Supplementary file 4 [file Table_2.DOCX]

**Supplementary Table 2.** N**umber of De novo varian**t**s fil**t**ered agains**t **ESP6500 and 1000** G**enomes** p**ublic varia**t**ion da**t**abases**

| **Single nucleotide**  **variants (SNVs)** | | Families | | | |  |  |
| --- | --- | --- | --- | --- | --- | --- | --- |
|  |  | **Family I** | **Family II** | **Family III** | **Family IV** | **Family V** | **Family VI** |
| **Total SNVs** | 4304 | | 5175 | 10590 | 7767 | 13650 | 12456 |
| **Non-synonymous SNVs** | 338 | | 497 | 854 | 741 | 967 | 1026 |
| **Filtered ESP6500** | 34 | | 38 | 48 | 51 | 173 | 126 |
| **Filtered Esp6500**  **and 1000 g** | 10 | | 20 | 16 | 22 | 68 | 42 |
