# Supplementary material for: Whole-Exome Sequencing of Discordant Monozygotic Twin Families for Identification of Candidate Genes for Microtia-Atresia
Source: Front Genet. 2020 Oct 22;11:568052. doi: 10.3389/fgene.2020.568052 (PMC7642525; doi:10.3389/fgene.2020.568052)
Supplement: Supplementary file 5 [file Table_3.DOCX]

**Supplementary Table 3. Go enrichment analysis revealled four gene sets and 11 related pathways**

| **Gene sets** | | **Pathways** | **Recurrent families** | **Recurrent times** |
| --- | --- | --- | --- | --- |
| DPY19L4/MUC6/MUC19/MUC4/NPC1/MUC16/MUC3A | | protein_glycosylation;macromolecule_glycosylation;glycosylation;glycoprotein_biosynthetic_process;glycoprotein_metabolic_process | TWS04_TWS02_TWS01_TWS03;TWS07_TWS08_TWS05_TWS06;TWS16_TWS17_TWS18_TWS19;TWS20_TWS21_TWS22_TWS23;XH1536_XH1537_XH1534_XH1535 | 5 |
| SPRR2F/KRTAP411/STRC/KRT26/KRTAP5-5/TRIOBP/HRNR | | epidermal_cell_differentiation;epidermis_development | TWS07_TWS08_TWS05_TWS06;TWS13_TWS15_TWS12_TWS14;TWS16_TWS17_TWS18_TWS19;TWS20_TWS21_TWS22_TWS23;XH1536_XH1537_XH1534_XH1535 | 5 |
| SPRR2F/KRTAP4-11/KRT26/KRTAP5-5/HRNR | | Keratinization;keratinocyte_differentiation;skin_development | TWS07_TWS08_TWS05_TWS06;TWS13_TWS15_TWS12_TWS14;TWS16_TWS17_TWS18_TWS19;TWS20_TWS21_TWS22_TWS23;XH1536_XH1537_XH1534_XH1535 | 5 |
| SCN8A/P2RX3/ITPR1/ATP1A1 | multicellular_organismal_signaling | | TWS04_TWS02_TWS01_TWS03;TWS13_TWS15_TWS12_TWS14;TWS16_TWS17_TWS18_TWS19;TWS20_TWS21_TWS22_TWS23 | 4 |
