# Supplementary material for: Whole-Exome Sequencing of Discordant Monozygotic Twin Families for Identification of Candidate Genes for Microtia-Atresia
Source: Front Genet. 2020 Oct 22;11:568052. doi: 10.3389/fgene.2020.568052 (PMC7642525; doi:10.3389/fgene.2020.568052)
Supplement: Supplementary file 6 [file Table_4.DOCX]

**Supplementary Table 4. Linkage-analysis combined with correlation-analysis revealed 13 related markers.**

| Name | Overtransmitted | T:U | Chi square | P value |
| --- | --- | --- | --- | --- |
| chr1_152186981_C_A | - | 0.042361111 | 0 | 1 |
| chr1_216011364_G_A | A | 0.083333333 | 2 | 0.1573 |
| chr3_69985838_G_A | A | 0.083333333 | 2 | 0.1573 |
| chr5_89949329_A_G | - | 0 | NaN | 0 |
| chr8_38287238_G_A | A | 0.041666667 | 1 | 0.3173 |
| chr8_72183989_G_A | A | 0.083333333 | 2 | 0.1573 |
| chr9_101546410_C_T | - | 0 | NaN | 0 |
| chr11_1018363_G_A | A | 0.16875 | 0.143 | 0.7055 |
| chr12_40882457_C_A | C | 0.16875 | 0.143 | 0.7055 |
| chr13_39338456_C_T | T | 0.083333333 | 2 | 0.1573 |
| chr17_38512919_G_T | T | 0.084027778 | 0.333 | 0.5637 |
| chr19_49344521_C_T | - | 0 | NaN | 0 |
| chr20_30072136_G_A | - | 0 | NaN | 0 |
